# The Effect of Thermal Pain on Working Memory: Behavioral Evidence from the n-Back Task

**DOI:** 10.3390/bs16050701

**Published:** 2026-05-04

**Authors:** Dong Dong, Koichi Hosomi, Nobuhiko Mori, Fanqing Zhou, Haruhiko Kishima, Youichi Saitoh

**Affiliations:** 1 Brain and Artificial Intelligence Laboratory, School of Automation, Northwestern Polytechnical University, 1 Dongxiang Road, Chang’an District, Xi’an 710129, China; 2 Department of Neurosurgery, Graduate School of Medicine, The University of Osaka, 2-2 Yamadaoka, Suita 565-0871, Osaka, Japan; k-hosomi@nsurg.med.osaka-u.ac.jp (K.H.); n-mori@nsurg.med.osaka-u.ac.jp (N.M.); hkishima@nsurg.med.osaka-u.ac.jp (H.K.); 3 School of Business, Xi’an International Studies University, South Wenyuan Road, Chang’an District, Xi’an 710128, China; zhoufanqing@xisu.edu.cn; 4 Tokuyukai Rehabilitation Clinic, 2-24-18 Shinsenri Nishimachi, Toyonaka 560-0083, Osaka, Japan; neurosaitoh@mbk.nifty.com

**Keywords:** thermal pain stimulation, working memory, cognitive load, n-back task

## Abstract

Pain and working memory interact bidirectionally, yet most paradigms treat pain as an extraneous distractor rather than task-relevant content. This study investigated whether thermal sensations can be encoded and updated in working memory in an n-back paradigm. Nineteen healthy adults completed visual (location) and thermal (temperature) single n-back tasks, cross-modal conditions with task-irrelevant distractors, and a dual n-back task across three load levels (1-, 2-, 3-back). Results showed that thermal cues consistently yielded lower accuracy and longer response times (RTs) compared to visual cues (*p* < 0.001, q < 0.001). While task-irrelevant thermal input tended to prolong RTs in the visual task under low load (*p* = 0.033, q = 0.099), task-irrelevant visual input showed a trend-level improvement in thermal-task accuracy under high load (3-back; *p* = 0.020, q = 0.060), consistent with a potential cross-modal effect. Qualitative data indicated that participants strategically transcoded thermal sensations into word or numerical labels to support maintenance. These findings demonstrate that pain can be operationalized as mnemonic content, though its processing incurs significant executive costs due to transcoding demands and resource competition. By shifting focus from “pain-as-interference” to “pain-as-content”, this paradigm offers a principled approach for the mechanistic study of nociceptive working memory updating and provides a foundation for quantifying cognitive interference in clinical pain populations.

## 1. Introduction

Pain and cognitive function have garnered increasing research interest, due to their intertwined influences on cognition and behavior. Working memory involves a limited amount of information in a temporarily heightened state of accessibility (Cowan, 2017) and supports executive functioning via prefrontal mechanisms that maintain task-relevant representations and cognitive control (Kimberg et al., 1997; Baddeley, 2003). Meanwhile, pain is not merely a sensory event, but also a complex phenomenon affecting emotional, cognitive, and motivational states (Melzack, 1999).

As a widely used paradigm for probing working memory updating and monitoring under load, the n-back task requires participants to continuously monitor a stimulus stream and respond when the current item matches one presented n items earlier. The n-back task effectively engages multiple components of working memory and has been utilized to explore the neural underpinnings of these processes (Owen et al., 2005). At the same time, the construct validity of the n-back task has been debated, with some accounts viewing it as reflecting broader working memory functioning, whereas others emphasize its closer ties to updating, monitoring, and attentional control (Jaeggi et al., 2010; Kane et al., 2007). This debate aligns with the broader view that working memory comprises multiple facets and task demands (Cowan, 2017). Accordingly, in the present study, we use the n-back task as an operational paradigm for examining how nociceptive versus visual information is maintained and updated under increasing cognitive load.

Moreover, evidence suggests that pain significantly impairs working memory. Attridge et al. (2015) demonstrated that both acute and chronic pain disrupt performance on the n-back task. Pain impairs working memory primarily by commandeering attentional resources needed for other tasks, as it shifts focus toward the body’s urgent need for escape and repair (Eccleston & Crombez, 1999). This cognitive resource competition can lead to decreased performance on tasks like the n-back (Moriarty et al., 2011). Moreover, prior work suggests that individual differences in pain perception and working memory performance, influenced by factors like pain catastrophizing, trait anxiety, and mindfulness, further modulate this effect (Tabry et al., 2020). These findings suggest that psychological traits and coping strategies play significant roles in the relationship between pain and working memory.

Although the disruptive effects of pain on working memory have been documented, several questions remain unanswered. For instance, the degree to which various pain types (e.g., acute vs. chronic) and differing levels of working memory load influence cognitive performance remain unclear. Meanwhile, interventions such as cognitive training and mindfulness are being explored for their potential to reduce pain’s impact on working memory (Jaeggi et al., 2010; Soveri et al., 2017). The interplay among cognitive, emotional, and neural processes underlies the relationship between pain and working memory. Studies using the n-back task have significantly advanced our understanding of pain’s cognitive costs and its influence on working memory vulnerability.

Previous studies have established a foundational understanding of the relationship between pain and working memory, yet they have typically treated pain as either a distracting influence or an independent state factor that modulates task performance (Boselie et al., 2016; Buhle & Wager, 2010; Lithfous et al., 2023; Nakae et al., 2013; Vogel et al., 2022). Research has rarely examined whether thermal sensations themselves can serve as the to-be-remembered content in a working memory updating task. Thermal stimulation provides a useful modality for this purpose because it can be delivered with precise experimental control and spans both painful (e.g., hot/cold nociceptive) and non-painful (warm/cool) sensory intensities, making it well suited for probing how bodily signals are encoded, maintained, and updated over time (Belmonte et al., 2009; Dubin & Patapoutian, 2010). Accordingly, the present study had three aims. First, we compared thermal and visual single n-back tasks across 1-, 2-, and 3-back cognitive loads to test whether updating performance differed by stimulus modality, expecting lower accuracy and longer response times for thermal cues. Second, we examined whether thermal input functioned differently when task-relevant versus task-irrelevant in cross-modal single task conditions, expecting task-irrelevant thermal input to disrupt visual performance, particularly under lower load, and task-irrelevant visual input to influence thermal-task performance under higher load. Third, we assessed the cost of concurrently monitoring nociceptive and visual information in a dual n-back task. Because the role of thermal information in dual-task updating remains unclear, analyses of this condition were treated as informative but exploratory.

## 2. Materials and Methods

### 2.1. Participants

During the recruitment phase of the experiment, participants were preliminarily screened based on their scores on the Short Form of Raven’s 2 (Raven’s 2 Progressive Matrices) (Langener et al., 2022). Those with Raven IQ scores significantly below the average for their age group were excluded because of their potential to have lower working memory performance (Unsworth & Engle, 2005). In this study, none of the participants had a Raven IQ below average in their respective age groups.

In addition, participants completed a brief health screening questionnaire at recruitment. Individuals were excluded if they reported any current or recurrent pain condition (acute or chronic), a history of clinically diagnosed pain-related disorders, ongoing medical evaluation or treatment for pain, regular use of analgesic or psychoactive medication, or a recent injury/trauma that could affect somatosensory or pain processing. Participants included in the study reported no relevant medical conditions, had not sought medical care for pain-related complaints, and reported no recent injury or trauma, thereby minimizing the likelihood of including individuals with acute or chronic pain.

Twenty individuals participated in this study. One participant was excluded owing to fatigue, which resulted in withdrawal from the experiment. Consequently, data from 19 participants (Table 1) aged between 22 and 28 years (mean age = 25, standard deviation [SD] = 1.5 years) were analyzed. Sample size was determined based on feasibility for an intensive within-subject, multi-condition protocol. To facilitate interpretation of statistical sensitivity, we report standardized within-subject effect sizes (Cohen’s d) for key contrasts and provide a sensitivity analysis indicating that, with *N* = 19 and α = 0.05 (two-tailed), the design has 80% power to detect within-subject effects of approximately d ≈ 0.68 (medium-to-large). All participants were in good physical health, did not take any medication, and had normal or corrected-to-normal vision. This study was approved by the Ethics Committee of Osaka University Hospital, and all participants provided written informed consent before inclusion in this study.

### 2.2. Instrument and Software

**Figure 1:**
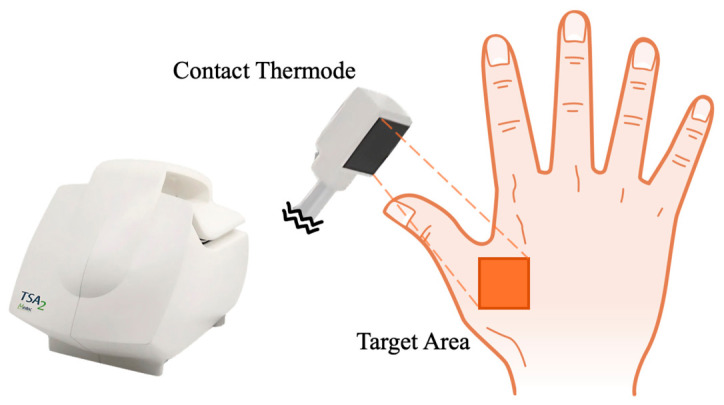
Thermal stimulation unit and target area for contact thermode placement. The main stimulation unit was a TSA2 (Medoc-TSA2 Advanced Thermosensory Stimulator, Israel; Medoc Ltd., 2019). The unit was connected to a contact thermode, and the temperature of the black square placed on the target area was manipulated.

For the presentation of stimuli and the recording of participant responses in the n-back tasks, a desktop computer equipped with a 24-inch monitor featuring a resolution of 1024 × 768 pixels was positioned 55 cm in front of the participant. The TSA2 (Medoc-TSA2 Advanced Thermosensory Stimulator, Ramat Yishai, Israel) was used to generate temperatures and apply stimulation to target areas. In addition to the main unit, a contact thermode measuring 30 mm × 30 mm was connected to apply thermal stimuli to the target area (Figure 1).

PsychoPy (version 2021.1.2) and dedicated software for TSA2, MMS (Medoc Main Station Version: 7.0.0.13.2), were used to create stimulus prompts and collect the experimental data. Specifically, visual stimuli were created using PsychoPy, whereas thermal stimuli were generated using MMS. Using PsychoPy’s Python (Version: 3.8.1) partial program, to access the External Control feature in MMS, we were able to conduct a series of tasks that included thermal stimuli in conjunction with visual stimuli. The performance scores and reaction times (RTs) were saved in real time during the tasks.

### 2.3. Stimuli

**Figure 2:**
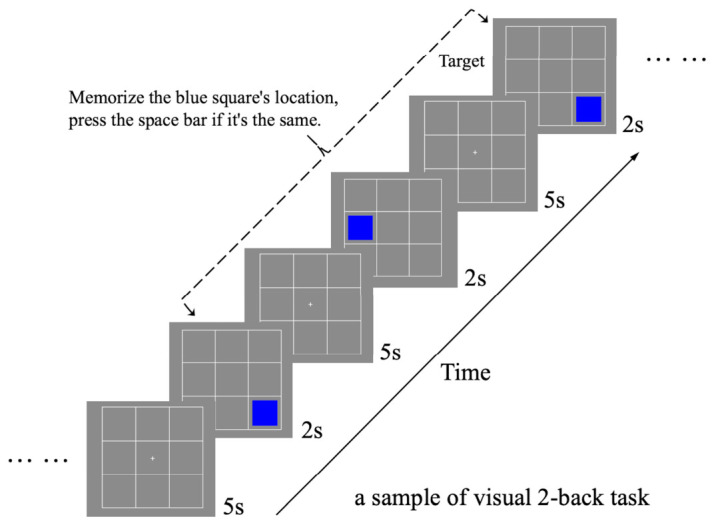
Visual n-back task. This is a sample of visual 2-back task, requiring participants to identify if the location of the blue square matches one presented “2” trails earlier in a sequence.

**Figure 3:**
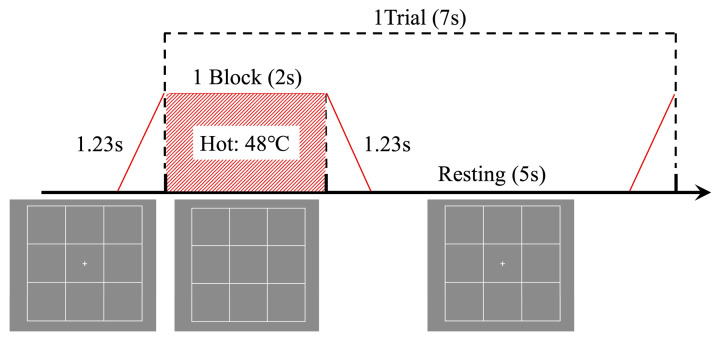
Thermal n-back task. The figure displays the thermal stimulus sequence in a trial, where the red diagonal lines show the temperature increase and decrease. Below the timeline are the visual stimuli observed by the participants during the task.

All tasks in this study were based on the standard n-back task, by requiring participants to identify if a current stimulus matches one presented “n” steps earlier in a sequence (Figure 2). During task execution, the display was configured to show a 3 × 3 grid in white (RGB: 1, 1, 1) centered on a gray background (RGB: 0, 0, 0). The task stimuli were categorized into two types: visual and thermal. Each trial lasted 7 s and consisted of a 2 s stimulus presentation period and a 5 s rest period. Depending on the task condition, the trial contained a visual stimulus, a thermal stimulus, or both. During the rest period, a “+” symbol was presented at the center of the screen to maintain participants’ focus. Visual stimuli were represented using blue squares (RGB: −1, −1, 1) within a 3 × 3 grid, facilitating mnemonic cues for the participants (Figure 2). Thermal stimuli were delivered via a contact thermode, connected to TSA2, and applied to the purlicue on the back of the hand (Figure 1). Thermal stimulation was initiated from a baseline temperature of 32 °C, with four distinct stimulus intensities (Belmonte et al., 2009; Dubin & Patapoutian, 2010; Salminen et al., 2013): strong stimuli (hot at 48 °C and cold at 16 °C), which are typically perceived as thermal pain stimuli, and mild stimuli (warm at 38 °C and cool at 26 °C). The transition from baseline to target temperatures occurred at the onset of stimulus presentation, with both heating and cooling durations precisely timed at 1.23 s, facilitating a controlled experimental environment for the assessment of thermal and visual stimuli integration. When the stimulus presentation period ended, the temperature transitioned from the target temperature back to the baseline, requiring an identical duration of 1.23 s (Figure 3). During the subsequent 5 s rest period within each trial, the thermode was maintained at the baseline temperature. During the inter-block rest period, the thermode was likewise held at 32 °C until participants reported no residual thermal sensation, thereby providing a perceptual reset and minimizing carryover effects.

### 2.4. General Procedure

The room temperature was maintained at 25 °C. The participants were asked which thumb they normally used to press the space bar while using a keyboard and were instructed to use the same thumb for all task responses during the experiment (Wilimzig et al., 2012). Subsequently, contact mode was maintained. Prior to the commencement of the tasks, the participants were subjected to temperatures of 48 °C and 16 °C for 5 s each, using the TSA2 devices. During this time, they were required to assess their pain sensations using the visual analog scale (VAS) once for the hot condition and once for the cold condition. For the exposures to 38 °C (warm) and 26 °C (cool), the participants were only asked to experience these temperatures without providing VAS assessments. The VAS consists of a horizontal line, 100 mm in length, anchored by two endpoints representing the extremes of “no pain/no feeling” and “worst imaginable pain/most severe discomfort.” The participants were instructed to indicate their current level of pain by marking a point on the line that best represented their perceived intensity at the time of assessment (Aitken, 1969; Delgado et al., 2018; Huskisson, 1974). Prior to the main task, all four target temperatures (48 °C, 38 °C, 26 °C, and 16 °C) were presented at least once (5 s per stimulus) for familiarization and a brief discriminability check. Participants verbally categorized each stimulus as “hot,” “warm,” “cool,” or “cold” and confirmed that the four sensations were distinguishable. If any difficulty was reported, additional practice exposures were administered until the participant indicated clear discriminability before proceeding. The experiment lasted approximately 1–1.5 h, varying with task completion pace.

Each n-back task comprised three levels of cognitive/memory load (Carlson et al., 1998; Jaeggi et al., 2010; Kane et al., 2007): 1-back (low load), 2-back (medium load), and 3-back (high load), with each run lasting 3.7 min (32 trials) at n-back level. The stimuli were arranged in a pseudo-random order to ensure balanced target location and temperature changes across the different runs. All runs were matched for the number of target (50%) and non-target (50%) occurrences. In the dual n-back task, the numbers of visual and thermal stimulus targets were equal. In addition to the dual n-back task, each task was performed once at the same load level. Each load level in the dual n-back task comprised two runs to maintain consistent target/non-target frequencies and probabilities with the other tasks.

**Figure 4:**
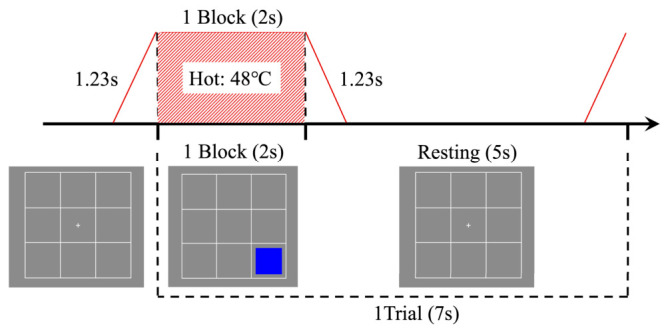
Stimulus presentation method of visual single n-back with thermal stimulation, thermal single n-back with visual stimulation, and dual n-back tasks. The figure displays the thermal stimulus sequence in a trial, where the red diagonal lines show the temperature increase and decrease. Below the timeline are the visual stimuli observed by the participants during the task.

The participants were required to complete the following five distinct experimental tasks:

1. Visual single n-back: Participants were instructed to remember the location of a blue square and judge whether it matched the location presented n trials earlier. They pressed the spacebar when the current square appeared in the same location as the square presented n trials earlier (Figure 2);
2. Thermal single n-back: Participants were instructed to remember the sensation of a temperature change and judge whether it matched the thermal sensation presented n trials earlier. They pressed the spacebar when the current thermal sensation matched the one presented n trials earlier (Figure 3);
3. Visual single n-back with thermal stimulation: Participants were instructed to remember the location of a blue square, as in the visual single n-back task, while disregarding the thermal stimulation. Thermal stimuli were presented pseudo-randomly with equal probability across trials. They pressed the spacebar when the current square appeared in the same location as the square presented n trials earlier (Figure 4);
4. Thermal single n-back with visual stimulation: Participants were instructed to remember the sensation of a temperature change while disregarding the location of the square. Visual stimuli were presented pseudo-randomly with equal probability across trials. They pressed the spacebar when the current thermal sensation matched the one presented n trials earlier (Figure 4);
5. Dual (visual and thermal) n-back: Participants were instructed to remember both the location of the square and the sensation of the temperature change and judge whether either dimension matched that presented n trials earlier. They pressed the spacebar when either the current square location or the current thermal sensation matched the corresponding stimulus presented n trials earlier, with the two types of target events arranged not to occur simultaneously (Figure 4).

The participants were advised to respond as quickly and accurately as possible using their dominant thumb to press the space bar for the target stimuli and not to respond to non-target stimuli. The task conditions were explained to the participants, followed by a practice trial. Ten participants started with the visual single n-back task and then proceeded to the thermal single n-back, followed by the visual single n-back with distractive thermal stimulation and thermal single n-back with distractive visual stimulation. The remaining participants performed the tasks in the reverse order: thermal single n-back, visual single n-back, thermal single n-back with distractive visual stimulation, and visual single n-back with distractive thermal stimulation. All the participants completed the experiment using a dual-n-back task. Within each type of task, runs commenced with the 1-back task, followed by the 2-back and 3-back tasks, with a 1–5 min rest period between tasks. Performance was evaluated based on RTs (for hits only) and accuracy scores (hits minus false alarms). After all tasks were completed, each participant was interviewed to document the strategies employed in n-back tasks involving thermal stimuli. Strategies for the visual task were not probed, as encoding strategies in the visual location n-back paradigm are already well characterized in the literature; therefore, the interviews focused on the novel thermal n-back task.

In the visual single n-back task with thermal stimulation, the participants were instructed to solely focus on memorizing visual stimuli without memorizing thermal stimuli. In the single thermal n-back with visual stimulation task, the focus shifts to memorizing the thermal stimuli, with no memorization of the visual stimuli required. In the dual n-back task, the participants were tasked with memorizing both visual and thermal stimuli.

### 2.5. Statistical Analysis

SPSS Statistics (version 29.0.2) was used for the analysis. Correlation analyses between accuracy and RT were conducted as an exploratory assessment of whether better task performance was associated with faster responding within each task/load condition, thereby providing descriptive information about potential speed–accuracy trade-offs. Pearson’s correlation coefficient was used for these analyses. Paired *t*-tests were used to compare accuracy and RTs across cognitive load conditions in the visual, thermal, and dual n-back tasks. Additional comparisons were made across modality (thermal vs. visual), presence of distractive stimulation, and thermal intensity (e.g., hot vs. cold). Two-tailed tests were conducted. Given the large number of comparisons, statistical significance was determined using FDR-adjusted q-values (q < 0.05). Results with q ≥ 0.05 and <0.10 were described as trend-level findings. Uncorrected *p*-values are reported for completeness.

## 3. Results

Participants had a mean age of 25 years (SD = 1.5). Of these, 7 (39%) were male, and 8 (42%) reported predominantly using their right thumb when pressing the space bar. The mean Raven IQ score was 104 (SD = 7.5). The VAS scores were significantly higher in hot conditions (mean VAS score = 88.3 mm, SD = 10.75) than in cold conditions (mean VAS score = 70.5 mm, SD = 12.29), with a mean difference of 17.79 (t = 5.38, SD = 14.47, *p* < 0.001) (Table 1). This suggests that the participants’ perception of hot temperature stimuli at 48 °C is more intense than that of the cold temperature stimuli at 16 °C.

Performance for each task under all conditions is shown in Table 2. Although performance was lower for thermal than for visual cues, participants still achieved meaningful accuracy across loads in the thermal n-back task (Accuracy (prop.): 1-back, 0.87; 2-back, 0.78; 3-back, 0.70), suggesting that thermal sensations could serve as reliable mnemonic cues within the n-back structure. The results of Pearson’s correlations showed a negative correlation between accuracy scores and RTs in the thermal single n-back task under the 1-back condition (r = −0.49, *p* = 0.032), indicating that a better score performance correlates with shorter RTs. No statistically significant correlations were found for the other n-back tasks across all conditions (Table A1).

**Figure 5:**
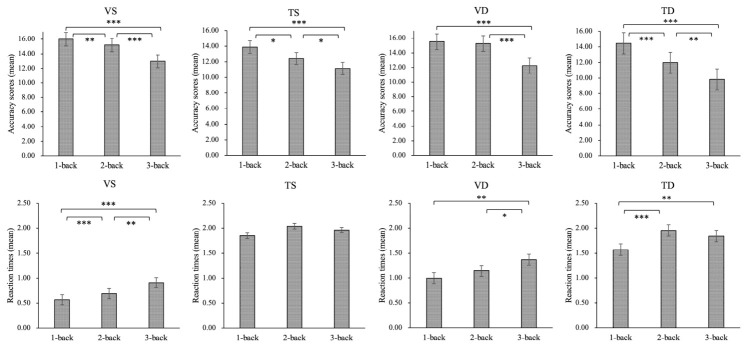
Paired *t*-test comparisons of visual single, thermal single, and dual n-back performance across cognitive load conditions. *: *p* < 0.05; **: *p* < 0.01; ***: *p* < 0.001; VS: visual single n-back task; TS: thermal single n-back task; VD: visual stimulus component in the dual n-back task; TD: thermal stimulus component in the dual n-back task.

To explore changes in task performance under various cognitive load conditions, comparisons were conducted for each pair of conditions (1-back vs. 2-back, 2-back vs. 3-back, and 1-back vs. 3-back) in both visual and thermal single n-back and dual n-back tasks (Figure 5, Table 3). Results summarized in Table 3 indicated that, in the visual single n-back and most components of the dual n-back tasks, higher cognitive load levels were associated with lower accuracy scores and longer RTs. However, in the thermal single n-back tasks, although higher cognitive load levels were associated with lower accuracy scores, RTs were not influenced by cognitive load levels (1- vs. 2-back, *p* = 0.288; 2- vs. 3-back, *p* = 0.585; 1- vs. 3-back, *p* = 0.342) (Figure 5, Table 3).

In terms of accuracy scores, the visual single n-back performance was superior to the thermal single n-back performance across all cognitive load levels (Table 4) (1-back, *p* < 0.001, q < 0.001; 2-back, *p* < 0.001, q < 0.001; 3-back, *p* < 0.001, q < 0.001). Visual single-n-back tasks exhibited shorter RTs than thermal single-n-back tasks across all cognitive load levels (Table 4) (1-back, *p* < 0.001, q < 0.001; 2-back, *p* < 0.001, q < 0.001; 3-back, *p* < 0.001, q < 0.001). In the dual n-back task, while visual and thermal stimuli were presented concurrently, participants were not required to respond to both simultaneously. Specifically, responses were prompted by either the visual or the thermal stimulus alone at any given time. This design allowed for separate analysis of the visual and thermal components. We compared performance in these components in terms of both accuracy and RTs (Table 4). These results were consistent with those obtained in the single n-back task, where visual stimuli outperformed thermal stimuli in terms of accuracy scores (1-back, *p* = 0.037, q = 0.037; 2-back, *p* < 0.001, q < 0.001; 3-back, *p* = 0.014, q = 0.021) and RTs (1-back, *p* < 0.001, q < 0.001; 2-back, *p* < 0.001, q < 0.001; 3-back, *p* < 0.001, q < 0.001).

We compared the visual single n-back with distractive thermal stimulation to the visual single n-back alone (Table 4), and found no statistically significant differences in accuracy scores or RTs after FDR correction. The RT difference in the 1-back condition was significant at the uncorrected level (*p* = 0.033) but did not survive FDR correction (q = 0.099) and was therefore treated as a trend-level effect. In the comparison between the thermal single n-back with distractive visual stimulation and the thermal single n-back alone (Table 4), the 3-back accuracy difference was significant at the uncorrected level (*p* = 0.020) but did not survive FDR correction (q = 0.060), indicating a trend-level advantage for the condition with task-irrelevant visual input. The corresponding RT difference (*p* = 0.040) did not survive FDR correction (q = 0.108).

To explore the effects of different temperature intensities on task performance in visual tasks, we analyzed performance under various temperature conditions in the single visual n-back with thermal stimulation, differentiating between hot vs. warm and cold vs. cool comparisons. We also analyzed conditions categorized as strong stimuli (average performance in hot and cold conditions) and mild stimuli (average performance in warm and cool conditions). The analysis revealed no statistically significant differences between the conditions across different temperature intensities (Table 5). We analyzed data from the thermal single n-back task under different cognitive load conditions. Hot stimuli achieved higher accuracy and shorter RTs than warm ones (except in the 3-back), while cold stimuli exhibited better accuracy than cool stimuli. No significant differences were observed between hot and cold stimuli (described in Table 6).

Our post-task interview records showed that, in the thermal single n-back tasks used in this study, 17 participants indicated that they needed to memorize perceived temperature as verbal cues (e.g., the word “hot”) to successfully perform the task. The other two participants used numbers as cues for temperature (e.g., using “4” to represent “hot”). In the dual n-back tasks, all participants reported relying on word labels for the thermal sensations during task execution.

## 4. Discussion

To our knowledge, this study is the first to implement an n-back task employing thermal stimuli as cues. The main result of our study was that thermal stimuli showed lower accuracy and longer RTs compared to visual stimuli. This effect was consistent across both single and dual n-back tasks, at all levels of cognitive load, and for both single and simultaneous stimulus presentations. The dual n-back condition extends the single-task findings by showing that the disadvantage for thermal information persists even when participants must monitor both sensory streams concurrently. This suggests that the cost associated with thermal cueing is not limited to isolated encoding demands, but generalizes to a context requiring concurrent monitoring of internal and external sensory information. Importantly, this pattern should not be interpreted as failure of the thermal task; rather, it indicates that although thermal information can be maintained and updated in working memory, doing so incurs greater executive costs than processing visual–spatial information. At the same time, the present findings should not be interpreted as exclusively pain-specific. Because the thermal task included both painful and non-painful temperature categories, the observed cost likely reflects a broader somatosensory/thermal encoding burden, to which pain-related salience may contribute but which is not reducible to nociception alone. Additionally, our interviews revealed that during the thermal single n-back tasks, all participants adopted either word-based or numerical cues to encode temperature information. During the dual n-back tasks, participants relied exclusively on word-based cues to encode the thermal sensations. These qualitative interview data provide a potential mechanistic explanation for the observed disparity. Participants consistently reported translating subjective thermal sensations into verbal or numerical labels (e.g., “level 2 hot”) to facilitate maintenance. In the framework of Baddeley’s working memory model (Baddeley, 1992; Baddeley, 2003), this suggests a strategic reliance on the phonological loop to compensate for the inherently transient and difficult-to-rehearse nature of thermal representations. In this sense, the additional cost observed in the thermal conditions may arise not only from pain-related salience, but also from the more general requirement to recode transient somatosensory input into a stable representational format for working memory maintenance. Unlike visual–spatial stimuli, which can be more directly maintained in the visuospatial sketchpad, thermal stimuli may require an additional transcoding step—from sensory intensity to linguistic symbols—thereby increasing the computational demand on the central executive. Consequently, the limited-capacity central executive must allocate additional attentional control to coordinate this cross-modal translation, leaving fewer resources available for efficient updating (Eccleston & Crombez, 1999; Moriarty et al., 2011). Accordingly, we propose that working memory processing for hot and cold thermal stimuli in this study likely involved an interaction between the visuospatial sketchpad and the central executive to assess perceived temperature, while simultaneously encoding the perceived thermal stimulus into linguistic information via the phonological loop to support working-memory-based task performance.

Previous studies have used similar methodologies to examine the effects of visual and auditory stimuli on working memory (Amon & Bertenthal, 2018; He et al., 2022), while others have explored the interaction between thermal/pain perception and working memory (Boselie et al., 2016; Buhle & Wager, 2010; Lithfous et al., 2023; Nakae et al., 2013; Vogel et al., 2022). However, no prior research has specifically investigated somatosensory thermal pain as the stimulus stream within an n-back paradigm. By comparing performance on the visual single n-back task under thermal stimulation with visual-only conditions, we found that random thermal stimuli tended to increase RTs under low cognitive load (1-back), whereas this interference was not evident under higher cognitive loads (2- and 3-back). Thus, the present findings speak most directly to load-dependent interference in n-back monitoring/updating performance, rather than to global working memory capacity.

At lower cognitive loads, participants may remain more perceptually and attentively sensitive to thermal pain, allowing task-irrelevant nociceptive input to capture attention and thereby prolong RTs; as cognitive load increases, resource-allocation strategies may shift toward task engagement, reducing sensitivity to the thermal signal. Importantly, this interpretation fits the broader bidirectional relationship between pain and cognition. Prior work has shown that engaging in demanding working memory tasks can attenuate pain perception—often referred to as cognitively induced analgesia (Paris et al., 2013; Buhle & Wager, 2010). Our findings complement this literature by highlighting the reciprocal cost: when nociceptive input is present, its biological salience (Eccleston & Crombez, 1999) can compete for executive control resources, producing a near “zero-sum” trade-off between task processing and pain processing. This framework provides a plausible account of why pseudo-random thermal stimulation increased RTs under low load (1-back) but not under higher loads (2- and 3-back), as stronger task engagement at higher loads may elicit greater top-down suppression of nociceptive processing, consistent with evidence linking working memory performance and pain modulation (Nakae et al., 2013). Neuroimaging evidence supports close interactions between pain processing and working memory, with n-back performance consistently engaging frontoparietal regions implicated in executive control, including prefrontal and parietal cortices (Owen et al., 2005). Building on this, Anderson et al. (2021) integrated neuroimaging with self-report measures of affective distress in healthy individuals and showed that pain is associated with poorer performance on working memory tasks, underscoring the broader coupling among nociception, cognition, and affect. Within this context, our cross-modal manipulation yields an additional and more nuanced insight: when comparing the thermal single n-back with task-irrelevant visual input to the thermal-only single n-back, we observed that the presence of a visual stream improved accuracy and shortened RT specifically under high load (3-back). We propose that, under maximal working memory demands, a stable visual stream may provide cross-modal scaffolding that facilitates the encoding and retrieval of thermal information—potentially by offering a “temporal anchor” that helps participants discretize, segment, and track fluctuating thermal sensations, consistent with interactions between top-down visual processing and working memory control (Kveraga et al., 2007). Importantly, this finding points to a potential translational implication for multimodal cognitive interventions: rather than relying solely on distraction, structured, non-painful sensory cues may help the brain manage nociceptive information more efficiently by supporting organization or “chunking” of pain-related representations during cognitively demanding situations.

Recent studies have increasingly highlighted the interplay between pain perception and cognition, demonstrating that cognitive interventions may alleviate pain and improve functioning (Higgins et al., 2018; Holzer et al., 2024; Khera & Rangasamy, 2021). For example, Holzer et al. (2024) reported that neurocognitive training targeting cognitive flexibility, memory, attention, and processing speed not only improved cognitive flexibility but also reduced pain severity relative to usual care, underscoring the clinical potential of targeted cognitive tasks and motivating closer examination of pain-related working memory processes. Building on this literature, our paradigm may serve as a candidate mechanism-oriented assessment tool. Whereas traditional cognitive tasks primarily index global cognitive decline or broad executive dysfunction, our thermal n-back variant assesses the efficiency of encoding, maintaining, and updating nociceptive information within a controlled working memory framework. This feature may help to operationalize and quantify the “cognitive interference” frequently reported by individuals with persistent pain, thereby informing more tailored neurocognitive rehabilitation strategies (Holzer et al., 2024; Khera & Rangasamy, 2021).

This study has several limitations. Although the within-subject design increases statistical power, the modest sample size may have limited our ability to detect smaller effects and to test moderators reliably. The design also minimized variability in age and individual differences, which may constrain generalizability. Another limitation is that subjective VAS ratings were collected for the painful hot (48 °C) and cold (16 °C) stimuli, but not for the non-painful warm (38 °C) and cool (26 °C) stimuli; collecting ratings for all four temperatures would provide a stronger check that perceived intensity aligned with the intended stimulus categories. Replication in larger samples, especially in clinical pain cohorts, is needed to test robustness and potential moderators. In addition, future studies combining this paradigm with functional magnetic resonance imaging (fMRI) may help clarify the neural overlap between pain processing and executive control and strengthen translational relevance.

## 5. Conclusions

This study introduces an n-back paradigm in which thermal sensations serve as task-relevant cues. Relative to visual–spatial cues, thermal cues imposed a substantial performance cost, reflected in lower accuracy and longer RTs. By contrasting conditions in which thermal input was task-relevant versus task-irrelevant, the findings refine the bidirectional view of pain-cognition interactions: under low cognitive load, task-extraneous thermal stimulation interfered with performance, whereas higher working memory load was associated with attenuated interference effects of task extraneous thermal stimulation on behavioral performance. Framing nociceptive input as mnemonic content rather than background distraction provides a principled approach for examining how nociceptive representations are maintained and updated under controlled working memory demands.

## Figures and Tables

**Table 1:** Characteristics of the study participants.

Characteristics	Mean/*n*	SD/*n*%
Age (years)	25.0	1.5
Sex (male)	*n* = 7	39%
Thumb (right) ^a^	*n* = 8	42%
Raven IQ	104.0	7.5
VAS (hot)	88.3	10.8
VAS (cold)	70.5	12.3

^a^: Thumb of the left or right hand accustomed to pressing the space bar; Raven IQ: Calculated from the scores obtained on Raven’s two progressive matrices; VAS (hot)/(cold): Visual analog scale scores for pain intensity induced by hot and cold stimuli.

**Table 2:** Performance for each task under all conditions.

Task Types	Conditions	Accuracy Scores		Reaction Times (s)	
		Accuracy (prop.)	SD	Mean	SD
VS	1-back	1	0.00	0.57	0.16
	2-back	0.95	1.13	0.69	0.19
	3-back	0.81	2.47	0.91	0.36
TS	1-back	0.87	1.70	1.85	0.82
	2-back	0.78	2.83	2.04	0.72
	3-back	0.70	2.81	1.96	0.53
VT	1-back	1.00	0.00	0.62	0.15
	2-back	0.96	1.02	0.69	0.19
	3-back	0.89	2.29	0.81	0.29
TV	1-back	0.92	1.11	1.70	0.51
	2-back	0.85	2.09	1.74	0.45
	3-back	0.80	2.66	1.73	0.49
VD	1-back	0.97	1.26	0.99	0.21
	2-back	0.95	1.19	1.14	0.35
	3-back	0.77	3.03	1.37	0.47
TD	1-back	0.90	2.04	1.57	0.38
	2-back	0.75	2.71	1.95	0.53
	3-back	0.62	3.48	1.84	0.38

Accuracy (prop.): indicates proportion correct and was calculated as the number of correct responses divided by the total number of response-required trials in each condition (range: 0–1); SD: standard deviation; VS: visual single n-back task; TS: thermal single n-back task; VT: visual single n-back task with thermal stimulation; TV: thermal single n-back with visual stimulation; VD: visual stimulus component in the dual n-back task; TD: thermal stimulus component in the dual n-back task.

**Table 3:** Paired *t*-test analysis in comparing visual single, thermal single, and dual n-back task performance under various cognitive load conditions.

Task Types	Conditions	Accuracy Scores				Reaction Times (s)			
		t	SD	*p*	q	t	SD	*p*	q
VS	1- vs. 2-back	3.03	1.13	0.007	0.007	−4.75	0.11	<0.001	<0.001
	2- vs. 3-back	4.48	2.15	<0.001	<0.001	−3.60	0.27	0.002	0.002
	1- vs. 3-back	5.29	2.47	<0.001	<0.001	−4.62	0.32	<0.001	<0.001
TS	1- vs. 2-back	2.33	2.76	0.032	0.032	−1.10	0.77	0.288	0.513
	2- vs. 3-back	2.55	2.16	0.020	0.031	0.56	0.61	0.585	0.585
	1- vs. 3-back	4.35	2.75	<0.001	<0.001	−0.98	0.51	0.342	0.513
VD	1- vs. 2-back	0.65	1.76	0.523	0.523	−2.07	0.32	0.053	0.053
	2- vs. 3-back	4.26	3.07	<0.001	<0.001	−2.81	0.08	0.012	0.018
	1- vs. 3-back	3.87	3.68	<0.001	<0.001	−3.34	0.49	0.004	0.012
TD	1- vs. 2-back	4.10	2.63	<0.001	<0.001	−3.97	0.42	<0.001	<0.001
	2- vs. 3-back	3.37	2.79	0.003	0.003	1.17	0.42	0.258	0.258
	1- vs. 3-back	6.64	3.04	<0.001	<0.001	−3.53	0.33	0.002	0.003

SD: standard deviation; *p*: *p*-value; q: *p*-value adjusted for multiple comparisons using the False Discovery Rate (FDR) correction; VS: visual single n-back task; TS: thermal single n-back task; VD: visual stimulus component in the dual n-back task; TD: thermal stimulus component in the dual n-back task.

**Table 4:** Paired *t*-test analysis across all types of tasks under identical cognitive load conditions.

Task Types	Conditions	Accuracy Scores				Reaction Times (s)			
		t	SD	*p*	q	t	SD	*p*	q
VS vs. TS	1-back	5.41	1.70	<0.001	<0.001	−7.31	0.76	<0.001	<0.001
	2-back	4.61	2.63	<0.001	<0.001	−8.78	0.67	<0.001	<0.001
	3-back	4.61	1.74	<0.001	<0.001	−8.61	0.53	<0.001	<0.001
VD vs. TD	1-back	2.25	2.04	0.037	0.037	−8.41	0.30	<0.001	<0.001
	2-back	4.61	3.01	<0.001	<0.001	−6.95	0.51	<0.001	<0.001
	3-back	2.73	3.86	0.014	0.021	−5.93	0.34	<0.001	<0.001
VT vs. VS *	1-back					2.31	0.01	0.033	0.099
	2-back	0.72	1.27	0.480	0.480	0.00	0.17	1.000	1.000
	3-back	1.86	2.71	0.079	0.159	−1.09	0.40	0.290	0.436
TV vs. TS	1-back	1.76	1.96	0.096	0.096	−0.84	0.76	0.410	0.410
	2-back	1.79	2.69	0.090	0.096	−1.91	0.68	0.072	0.108
	3-back	2.56	2.69	0.020	0.060	−2.21	0.46	0.040	0.108

* Due to the ceiling effect, participants achieved full scores in both tasks in the 1-back condition, rendering the comparison inconclusive. SD: standard deviation; *p*: *p*-value; q: *p*-value adjusted for multiple comparisons using the False Discovery Rate (FDR) correction; VS vs. TS: comparing the visual single n-back task to the thermal single n-back task; VD vs. TD: comparing visual stimuli components to thermal stimuli components in the dual n-back task; VT vs. VS: comparing the visual single n-back task with thermal stimulation to the visual single n-back task; TV vs. TS: comparing the thermal single n-back with visual stimulation to the thermal single n-back task.

**Table 5:** Performance in the visual single n-back task under different thermal stimulation conditions.

		Accuracy Scores		Reaction Times (s)	
Conditions	Contrasts	t	*p*	t	*p*
1-back *	Hot vs. Warm			1.42	0.174
	Cold vs. Cool			0.24	0.817
	Strong vs. Weak			0.99	0.334
2-back	Hot vs. Warm	−0.89	0.384	−0.12	0.903
	Cold vs. Cool	0.81	0.429	0.29	0.772
	Strong vs. Weak	0.06	0.952	0.02	0.983
3-back	Hot vs. Warm	−0.26	0.800	−0.96	0.350
	Cold vs. Cool	0.22	0.829	1.34	0.197
	Strong vs. Weak	−0.06	0.954	−0.18	0.861

* Due to the ceiling effect, participants achieved full scores in both tasks in the 1-back condition, rendering the comparison inconclusive.

**Table 6:** Paired *t*-test analysis in the thermal single n-back.

		Accuracy Scores			Reaction Times (s)		
Conditions	Contrasts	t	*p*	q	t	*p*	q
1-back	Hot vs. Warm	2.36	0.029	0.069	−3.07	0.007	0.021
	Cold vs. Cool	2.14	0.046	0.069	1.11	0.281	0.422
	Hot vs. Cold	−0.62	0.542	0.542	0.82	0.424	0.424
2-back	Hot vs. Warm	3.38	0.003	0.008	−2.02	0.006	0.018
	Cold vs. Cool	3.24	0.005	0.008	0.56	0.579	0.750
	Hot vs. Cold	0.32	0.749	0.749	0.32	0.750	0.750
3-back	Hot vs. Warm	0.75	0.465	0.465	−3.92	0.001	0.003
	Cold vs. Cool	2.58	0.002	0.006	−1.71	0.106	0.159
	Hot vs. Cold	−0.97	0.344	0.465	1.48	0.159	0.159

*p*: *p*-value; q: *p*-value adjusted for multiple comparisons using the False Discovery Rate (FDR) correction; Explanation: To compare the performance differences among stimuli with varying temperature intensities, we analyzed data from the thermal single n-back task under different cognitive load conditions. The results showed that, except for the accuracy scores in the 3-back condition, hot stimuli outperformed warm stimuli by yielding higher accuracy scores and shorter reaction times (RTs). The comparison between cold and cool stimuli revealed that, across all conditions, cold stimuli had better accuracy scores than cool stimuli, although RTs did not differ significantly. Moreover, the comparison between hot and cold stimuli showed no statistically significant performance differences in any condition.

## Data Availability

The data presented in this study are available on request from the corresponding author. The data are not publicly available due to ethical restrictions.

## Abbreviations

The following abbreviations are used in this manuscript:

IQ	Intelligence quotient
SD	Standard deviation
RTs	Reaction times
VAS	Visual analog scale
FDR	False discovery rate

## Appendix A

**Table A1:** Correlations between accuracy scores and reaction times for each task under all conditions.

Task Types	Conditions	Pearson’s Correlations Between Accuracy Scores and Reaction Times	
		r	*p*
VS *	1-back		
	2-back	−0.16	0.517
	3-back	−0.43	0.066
TS	1-back	−0.49	0.032
	2-back	−0.37	0.122
	3-back	−0.20	0.408
VT *	1-back		
	2-back	−0.13	0.584
	3-back	−0.28	0.242
TV	1-back	−0.24	0.323
	2-back	0.01	0.975
	3-back	−0.11	0.652
VD	1-back	−0.16	0.512
	2-back	0.17	0.492
	3-back	−0.45	0.051
TD	1-back	0.27	0.256
	2-back	0.00	0.999
	3-back	−0.33	0.169

* Because participants reached ceiling performance in the 1-back condition, accuracy scores lacked sufficient variance (constant), precluding the calculation of correlations between accuracy scores and reaction times. VS: visual single n-back task; TS: thermal single n-back task; VT: visual single n-back task with thermal stimulation; TV: thermal single n-back with visual stimulation; VD: visual stimulus component in the dual n-back task; TD: thermal stimulus component in the dual n-back task.
